# Linking teacher caring to perceived course-related learning gains: the sequential mediating roles of academic self-efficacy and learning engagement

**DOI:** 10.3389/fpsyg.2026.1901610

**Published:** 2026-07-16

**Authors:** Peitao Du, Xiaoling Li, Xujin Xian

**Affiliations:** 1 Al-Farabi Kazakh National University, Almaty, Kazakhstan; 2 The Catholic University of Korea, Bucheon, Republic of Korea

**Keywords:** academic motivation, academic self-efficacy, learning engagement, perceived course-related learning gains, sequential mediation, teacher caring

## Abstract

Teacher caring is a crucial relational resource for student development in higher education, yet the process by which it connects to students’ perceived course-related learning gains remains underexplored. Drawing on caring theory, social cognitive theory, learning engagement theory, and a supportive relationship perspective, this study tested a sequential mediation model. This model posits that teacher caring is associated with perceived course-related learning gains through academic self-efficacy and learning engagement. A cross-sectional, anonymous online questionnaire survey was administered to Chinese undergraduate students. Out of 632 returned questionnaires, 562 valid responses remained after data cleaning, resulting in a valid response rate of 88.9%. The results showed that teacher caring was positively associated with academic self-efficacy, learning engagement, and perceived course-related learning gains. The CFA measurement model demonstrated a good fit, and composite score path analyses using standardized scores supported the proposed model. Bootstrap mediation tests indicated that both academic self-efficacy and learning engagement partially mediated the association between teacher caring and perceived course-related learning gains, and they also formed a significant sequential indirect path. These results remained stable even after controlling for gender, year of study, major category, region, and university identifier. The findings suggest that teacher caring should be understood not merely as emotional warmth or general support, but as a relational resource linked to students’ confidence in learning and active engagement in coursework. Because the study relied on cross-sectional self-report data, the results should be interpreted as statistical associations consistent with the proposed relational–motivational pathway rather than as evidence of causal ordering.

## 1 Introduction

In contemporary higher education, quality-oriented teaching reforms increasingly emphasize not only students’ final academic performance but also their perceived gains in knowledge understanding, competence development, learning strategies, autonomous learning, and developmental clarity during coursework (Chow et al., 2020; Ilie et al., 2025; Pampaka et al., 2018). Therefore, perceived course-related learning gains refer not simply to course grades or credit accumulation, but to whether students perceive meaningful growth through the learning process (Kuh, 2001; Parker, 2018). Student-centered teaching reform emphasizes support, feedback, and participation, requiring universities to attend not only to what is taught but also to whether students develop sustainable learning capacities through course learning (Freeman et al., 2014; Stains et al., 2018; Theobald et al., 2020).

Teachers are among the university members with whom students interact most directly. Their caring behaviors may therefore function as proximal relational resources that shape student learning and development (Kahu, 2013; Kahu and Nelson, 2018). Teacher caring commonly includes emotional support, academic support, respect and inclusion, and developmental guidance (Song et al., 2022; Teven and McCroskey, 1997; Wu and Cai, 2025). It involves teachers’ responses to students’ learning pressure and emotional states, specific guidance for learning difficulties, equal acceptance of students’ views, and support for students’ developmental direction.

Teacher caring is more than an expression of warmth; it is an educational relational practice offering emotional, cognitive, and developmental support (Noddings, 2012). Noddings (2012) theory emphasizes that caring in education is not an abstract moral principle but a relationship of response, acceptance, and responsibility embedded in concrete interactions. Similarly, Teven and McCroskey’s (1997) research indicates a strong association between students’ perceived teacher caring and their learning experiences and evaluations of teaching.

Prior studies generally agree that positive teacher–student relationships, teacher support, and quality classroom interaction are positively linked to students’ learning attitudes, engagement, and academic outcomes (Prananto et al., 2025; Quin, 2017; Roorda et al., 2011). However, identifying a positive association between teacher caring and learning outcomes is insufficient to clarify the statistical pathway linking the two. For university students, external support does not automatically translate into perceived course-related learning gains. Instead, teachers’ encouragement, feedback, and respect may first be internalized into students’ judgments about their own learning capability, which then reflects in behavioral, emotional, and cognitive states of sustained engagement in learning tasks (Bandura, 1977; Fredricks et al., 2004; Schunk and DiBenedetto, 2020). In other words, the key to the association between teacher caring and perceived course-related learning gains may lie not simply in students feeling cared for, but in whether such caring fosters the belief, “I can learn well,” and a learning state characterized by willingness and ability to remain engaged.

Based on this logic, the present study employs self-efficacy theory, learning engagement theory, and a supportive relationship perspective as supplementary explanatory lenses. It develops a statistical sequential mediation model linking teacher caring to perceived course-related learning gains (Huang and Wang, 2023; Wang et al., 2024; Zhao et al., 2024). Four research questions guide the study: First, is teacher caring significantly and positively associated with undergraduate students’ perceived course-related learning gains? Second, does academic self-efficacy mediate the relationship between teacher caring and perceived course-related learning gains? Third, does learning engagement function as a mediator? Fourth, do academic self-efficacy and learning engagement jointly constitute a sequential path from teacher caring to perceived course-related learning gains?

This study offers three potential contributions. First, it advances teacher caring research from outcome association to statistical pathway explanation by examining how teacher caring links to perceived course-related learning gains within a theoretically specified mediation model. Second, it integrates academic self-efficacy and learning engagement into the same model, clarifying how supportive teacher–student relationships associate with psychological resources and learning states (Kahu et al., 2020; Kahu and Nelson, 2018). Third, by drawing on the logic of supportive relationships, psychological resources, and learner role engagement, this study provides empirical implications for faculty development and student support systems in higher education (Kahn, 1990; Saks, 2006).

## 2 Literature review and hypotheses

### 2.1 Theoretical integration: from supportive relationships to perceived course-related learning gains

This section integrates caring theory, social cognitive theory, and learning engagement theory to establish the theoretical foundation for the subsequent hypotheses (Bandura, 1977; Fredricks et al., 2004; Noddings, 2012).

Caring theory offers a relational basis for understanding teacher caring. Noddings (2012) posits that educational caring centers on the response of the one-caring to the cared-for, emphasizing listening, understanding, acceptance, and supportive actions. In a university context, teacher caring is not merely a friendly attitude; rather, it is a supportive relationship perceived by students and embedded in teaching practices (Teven and McCroskey, 1997; Wu and Cai, 2025). Through classroom interaction, feedback, evaluative language, and developmental guidance, teacher caring shapes students’ sense of safety, belonging, and accessibility to help within the learning environment (Ruzek et al., 2016; Tao et al., 2022; Wentzel, 1997).

Social cognitive theory further explains how external support translates into individual action. Bandura (1977) defines self-efficacy as individuals’ judgments about their capacity to organize and execute actions required to achieve goals. These judgments influence goal choice, effort, persistence, and emotional responses to challenges (Pajares, 1996; Schunk and DiBenedetto, 2020). Given that university learning tasks often involve considerable autonomy and uncertainty, students’ belief in their ability to complete tasks and overcome difficulties is an important psychological link between external support and learning outcomes (Honicke and Broadbent, 2016; Zimmerman, 2000).

Learning engagement theory emphasizes that learning outcomes are not an automatic product of available resources; rather, they are realized through students’ actual participation in the learning process (Kahu and Nelson, 2018; Li and Xue, 2023). Fredricks et al. (2004) conceptualize engagement as behavioral, emotional, and cognitive. Kahu (2013) further notes that student engagement in higher education involves both individual psychological states and contextual influences, including the teaching environment, institutional support, and interactional relationships. Thus, teacher caring, academic self-efficacy, and learning engagement can be understood as a continuous chain of conversion, moving from supportive relationships to psychological resources and then to learning processes (Handelsman et al., 2005; Skinner et al., 2008; Wang et al., 2022).

### 2.2 Teacher caring and perceived course-related learning gains

Teacher caring refers to students’ perceptions of teachers’ emotional support, academic support, respect and inclusion, and developmental guidance during the learning process (Noddings, 2012; Teven and McCroskey, 1997; Wu and Cai, 2025). Emotional support involves teachers’ attention to students’ learning pressure, emotional states, and sense of acceptance. Academic support involves specific feedback, task clarification, and guidance on learning methods. Respect and inclusion involve teachers’ acceptance of students’ viewpoints and equitable responses in classroom interaction. Developmental guidance refers to teachers’ assistance in helping students clarify learning goals and future directions. Perceived course-related learning gains refer to students’ perceived growth in knowledge, competence, learning strategies, autonomous learning, and developmental clarity throughout coursework (Chow et al., 2020; Ilie et al., 2025; Parker, 2018).

From an educational psychology perspective, teacher caring may foster a stronger sense of safety and belonging in the learning environment among students. This, in turn, can lead to a greater willingness to disclose confusion, seek feedback, and actively participate in learning activities. From a supportive relationship perspective, teacher caring functions as a crucial proximal support resource in higher education. It may reduce uncertainty when students encounter learning tasks, making them more likely to interpret teacher feedback as a developmental resource rather than an evaluative threat (Jang et al., 2010; Nicol and Macfarlane-Dick, 2006; Wisniewski et al., 2020). Empirical studies consistently demonstrate associations between teacher support, positive teacher–student relationships, student engagement, and achievement (Roorda et al., 2011; Roorda et al., 2017; Skinner and Belmont, 1993). Therefore, students who perceive stronger teacher caring are more likely to have positive learning experiences and report higher perceived course-related learning gains (Prananto et al., 2025; Song et al., 2022; Tao et al., 2022).

*H1*: Teacher caring is positively associated with undergraduate students’ perceived course-related learning gains.

### 2.3 The mediating role of academic self-efficacy

Academic self-efficacy refers to students’ judgments about their ability to complete learning tasks, cope with learning difficulties, and make academic progress. Self-efficacy theory posits that individuals’ judgments of their own capability influence their goal choices, effort, persistence, and responses to setbacks (Bandura, 1977; Schunk and DiBenedetto, 2020). In academic contexts, self-efficacy is closely related to learning motivation, self-regulation, strategy use, and academic performance (Honicke and Broadbent, 2016; Pajares, 1996; Zimmerman, 2000).

In university coursework, teacher caring may enhance students’ academic self-efficacy in several ways. Teacher encouragement can reduce excessive fear of failure, specific feedback can help students identify pathways for improvement, and respect and inclusion can make students more willing to express themselves and attempt difficult tasks. Furthermore, developmental guidance can help students connect short-term learning tasks with long-term developmental goals (Jang et al., 2010; Nicol and Macfarlane-Dick, 2006; Wisniewski et al., 2020). Meanwhile, students with higher academic self-efficacy are more likely to actively approach complex tasks, persist when encountering difficulties, and transform feedback into strategy adjustments. Such capability beliefs influence not only learning behavior but also how students interpret learning outcomes. Thus, teacher caring may be linked to higher perceived course-related learning gains through stronger academic self-efficacy (Chemers et al., 2001; Huang and Wang, 2023; Zhao et al., 2024).

*H2*: Academic self-efficacy mediates the relationship between teacher caring and perceived course-related learning gains.

### 2.4 The mediating role of learning engagement

Learning engagement typically includes behavioral, emotional, and cognitive engagement. Behavioral engagement refers to classroom participation, task completion, and sustained effort. Emotional engagement refers to interest, positive emotions, and a sense of belonging. Cognitive engagement refers to deep thinking, strategy use, and self-regulation (Handelsman et al., 2005; Skinner et al., 2008; Yakup and Boyacı, 2021). Learning engagement theory posits that students’ learning outcomes depend on the time, energy, and psychological resources they invest in learning activities. This investment, in turn, is shaped by teacher support, classroom structure, and the perceived meaningfulness of learning (Fredricks et al., 2004; Kuh, 2001; Li and Xue, 2023).

Teacher caring may foster sustained student participation in learning by cultivating a supportive classroom climate, improving feedback quality, and strengthening students’ sense of respect (Ruzek et al., 2016; Wang et al., 2022; Zhou et al., 2022). Learning engagement directly links the learning process to learning outcomes. Even with ample external resources, stable perceived course-related learning gains are unlikely without actual participation and deep processing. Conversely, when students are behaviorally, emotionally, and cognitively engaged, learning tasks are more likely to result in improved knowledge understanding, competence development, and a clearer developmental direction (Carini et al., 2006; Kahu et al., 2020; Prananto et al., 2025). Thus, learning engagement may be an important process variable through which teacher caring is associated with perceived course-related learning gains.

*H3*: Learning engagement mediates the relationship between teacher caring and perceived course-related learning gains.

### 2.5 The sequential mediating role of academic self-efficacy and learning engagement

Academic self-efficacy and learning engagement are not independent psychological processes. Higher academic self-efficacy can strengthen students’ sense of control over learning tasks, making them more willing to invest time, emotion, and cognitive resources (Schunk and DiBenedetto, 2020; Zimmerman, 2000). Learning engagement then translates these capability beliefs into concrete learning processes and is statistically linked to perceived course-related learning gains. Previous research indicates that students’ self-efficacy is closely related to intrinsic value, cognitive engagement, and learning performance, and that student engagement is associated with multiple learning outcomes (Carini et al., 2006; Huang and Wang, 2023; Pintrich and De Groot, 1990).

Therefore, teacher caring may first be associated with stronger student capability beliefs through support and feedback, subsequently leading to greater learning engagement through these beliefs, and ultimately resulting in higher perceived course-related learning gains (Wu et al., 2020; Wu and Cai, 2025; Zhao et al., 2024). This logic aligns with the explanatory chain in organizational psychology, which moves from supportive relationships to psychological resources, role engagement, and outcomes (Kahn, 1990; Saks, 2006). In higher education, teacher caring represents a supportive relationship, academic self-efficacy constitutes a learning-related psychological resource, and learning engagement reflects students’ actual investment in their learner role (Kahu and Nelson, 2018; Kahu et al., 2020).

**Figure 1:**
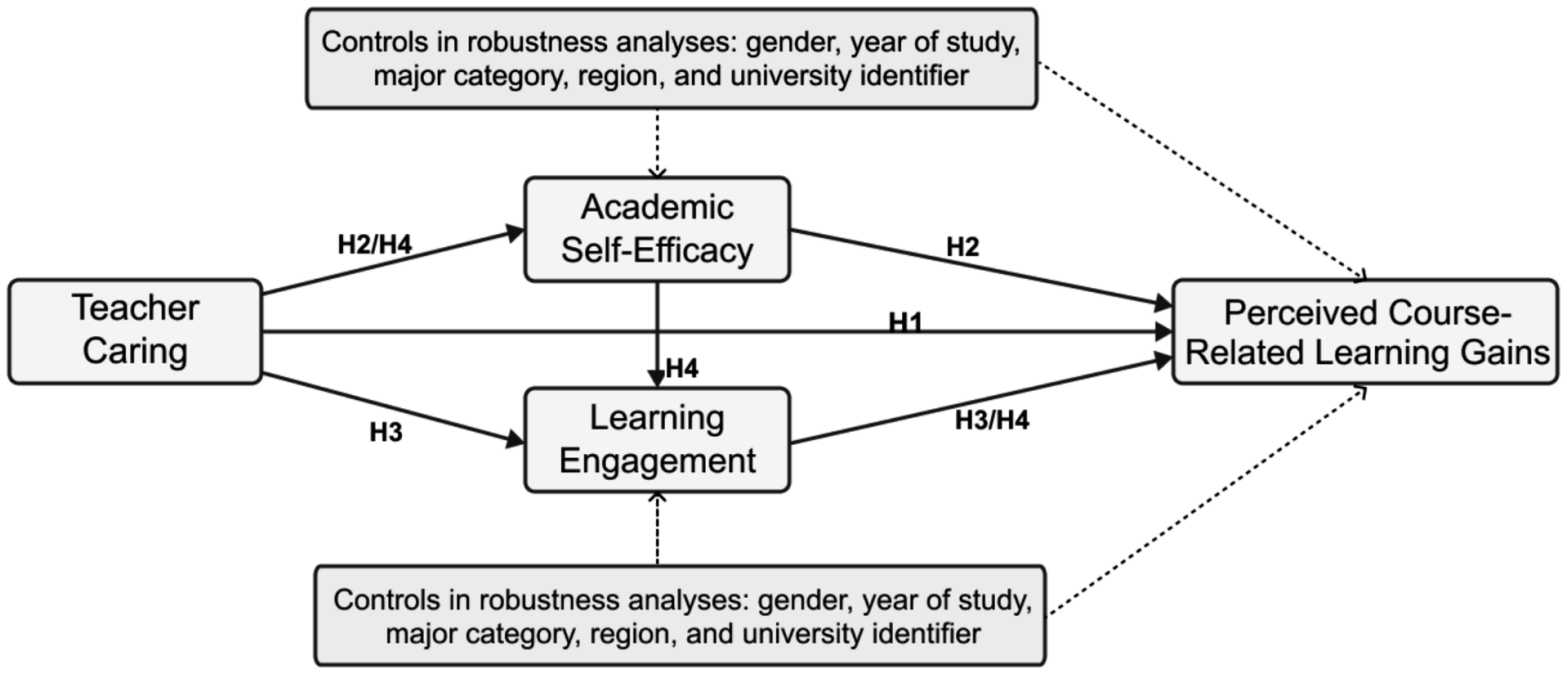
Research model and hypothesized paths. Solid arrows indicate hypothesized focal associations; dashed arrows indicate control-variable adjustments in robustness analyses. For visual clarity, the same set of control variables is displayed in two boxes; these boxes refer to the same robustness-adjustment variables rather than two separate sets of controls. H1 indicates the total association; H2 and H3 indicate separate mediating paths; H4 indicates the sequential mediation path.

*H4*: As illustrated in Figure 1, academic self-efficacy and learning engagement sequentially mediate the relationship between teacher caring and perceived course-related learning gains. Specifically, teacher caring is associated with perceived course-related learning gains, first through academic self-efficacy and then through learning engagement.

## 3 Methods

### 3.1 Sample and procedure

This study used a cross-sectional, anonymous online questionnaire survey administered to Chinese undergraduate students. The questionnaire was administered between April and May 2026 via an online survey platform. Participants were adult undergraduate students voluntarily recruited through university course groups and online student communities. To protect institutional confidentiality and reduce the identifiability of participating institutions, universities were anonymized as U1–U6. Before beginning the survey, participants were informed about the academic purpose of the study, questionnaire content, voluntary participation, anonymity, academic use of data, data protection, and their right to withdraw before submission. Completion and submission of the anonymous questionnaire were considered informed consent. The questionnaire included demographic information, measures of teacher caring, academic self-efficacy, learning engagement, perceived course-related learning gains, and attention-check items (Oppenheimer et al., 2009; Meade and Craig, 2012; DeSimone et al., 2015; Curran, 2016). A total of 632 questionnaires were returned. After data cleaning, 562 valid responses were retained, resulting in a valid questionnaire retention rate of 88.9%. The final data cleaning criteria included failed attention checks, overly short response times, obvious straight-lining, random response patterns, and near-duplicate responses (Curran, 2016; DeSimone et al., 2015; Meade and Craig, 2012). For the attention-check criterion, a response was classified as failed only if both attention-check items were answered incorrectly; responses that failed only one attention-check item were evaluated in conjunction with other response quality indicators rather than being excluded solely on that basis. For reporting purposes, exclusion categories were coded hierarchically and presented as mutually exclusive; if a response met multiple exclusion criteria, it was counted only once according to the first applicable criterion in the cleaning sequence.

Of the 562 valid responses, 318 participants (56.6%) were female and 244 (43.4%) were male. In terms of year of study, 159 were first-year students (28.3%), 175 were second-year students (31.1%), 147 were third-year students (26.2%), and 81 were fourth-year students (14.4%). Regarding major categories, 194 students (34.5%) were in humanities and social sciences, 190 (33.8%) in science and engineering, 109 (19.4%) in business and management, and 69 (12.3%) in education and other fields. For reporting and control-variable coding, smaller major categories were combined into the “education and other fields” category. In terms of region, 265 students (47.2%) were from central China and 297 (52.8%) were from eastern China. The region variable was coded according to these two regions represented in the final analytic sample. To protect institutional information, the dataset retained only university identifiers from U1 to U6. The stepwise data cleaning procedure that yielded the final valid sample is summarized in Table 1.

**Table 1:** Sample cleaning procedure.

Stage	Sample size	Description
Initial sample	632	All returned questionnaires
Excluded responses	70	Failed both attention checks = 16; overly fast completion = 14; straight-lining = 14; random response pattern = 14; near-duplicate responses = 12
Valid sample	562	Responses included in the formal statistical analysis

Exclusion categories were coded hierarchically and reported as mutually exclusive. If a response met multiple exclusion criteria, it was counted only once according to the first applicable criterion in the cleaning sequence.

A sample-size justification for the planned sequential mediation model was conducted using an *a priori* power analysis framework for mediation models (Schoemann et al., 2017). To avoid relying on effects estimated from the present data, the analysis assumed conservatively small standardized component paths for the focal sequential indirect association: teacher caring to academic self-efficacy (a1 = 0.15), academic self-efficacy to learning engagement (d21 = 0.15), and learning engagement to perceived course-related learning gains (b2 = 0.15), corresponding to a sequential indirect effect of 0.0034. The remaining direct paths in the model were set to small values, with standardized coefficients ranging from 0.10 to 0.15. With alpha = 0.05 and target power = 0.80, Monte Carlo simulations indicated that a minimum sample size of approximately N = 540 was required to detect the sequential indirect association using a two-sided 95% confidence interval. Therefore, the final analytic sample of N = 562 exceeded the estimated minimum requirement, providing sufficient statistical power for the hypothesized sequential mediation model. The assumptions used in the power analysis are summarized in Supplementary material S5.

### 3.2 Measurement, scale sources, and adaptation process

The teacher caring scale comprised 12 items across four dimensions: emotional support, academic support, respect and inclusion, and developmental guidance. Item development was informed by Noddings’ (2012) caring theory and Teven and McCroskey’s (1997) research on instructional caring. Items were contextualized to reflect specific manifestations of caring, feedback, respect, and developmental guidance in university classrooms (Song et al., 2022; Wu and Cai, 2025).

Academic self-efficacy was treated as a unidimensional construct, measured with six items. These items assessed students’ confidence in completing learning tasks, coping with learning difficulties, preparing for course assessments, and achieving learning goals. Item development drew on Bandura’s (1977) self-efficacy theory, Pajares (1996) research on academic self-efficacy, and Chemers et al.’s (2001) work on academic self-efficacy and university student adjustment (Honicke and Broadbent, 2016; Pampaka et al., 2018; Zimmerman, 2000).

Learning engagement comprised three dimensions: behavioral, emotional, and cognitive engagement, and was measured with nine items. Behavioral engagement focused on timely task completion and active participation; emotional engagement on learning interest, positive emotions, and perceived meaning; and cognitive engagement on connecting knowledge, understanding complex content, and reflecting on learning strategies (Handelsman et al., 2005; Skinner et al., 2008; Yakup and Boyacı, 2021). Item development was guided by Fredricks et al.'s (2004) three-dimensional engagement framework, Kahu’s (2013) theory of student engagement in higher education, and Handelsman et al.'s (2005) measurement of college student course engagement. Because the present study focused on the overall mediating role of learning engagement, the nine items were averaged into a composite score for the main analyses. This decision was based on the theoretical treatment of engagement as an overarching learning process construct and methodological guidance indicating that summated or averaged scale scores are appropriate when items are designed to represent a common construct and reliability/factor analytic evidence supports the score (Spector, 1992; Hinkin, 1998; DeVellis and Thorpe, 2021). The CFA specified learning engagement as one overall factor, and the reliability and validity results supported the use of the global score. Therefore, while the three dimension scores were retained for scoring transparency and potential supplementary description, dimension-specific mediation paths were not tested in the main model.

The questionnaire in this study was not a word-for-word translation of a single English scale, nor were established instruments mechanically converted from their original response formats. Instead, it was developed and adapted for the Chinese university context based on mature theories and established measurement dimensions. This adaptation involved four steps: Identifying and retaining core constructs and dimensions consistently present in prior research; adapting item wording to a learning-context language understandable to Chinese undergraduates. Self-report phrasing such as “I perceive,” “I believe,” and “I would,” while avoiding multiple judgment objects within a single item; and adopting a five-point Likert response format for all items, where 1 indicates “strongly disagree” and 5 indicates “strongly agree” (Beaton et al., 2000; Hinkin, 1998). The five-point response format was integral to the instrument’s design; therefore, this study does not claim direct measurement equivalence with any original instrument that uses a different response format. As independent pilot test raw records were not available in the existing materials, no separate pilot sample or cross-format measurement invariance analysis is reported. To ensure the validity of the adapted five-point instrument, measurement quality was evaluated through semantic appropriateness, data cleaning, reliability analysis, convergent validity, discriminant validity, approximate HTMT, and the CFA measurement model (Fornell and Larcker, 1981; Henseler et al., 2015; Hu and Bentler, 1999). Further details on demographic coding, measurement items, data cleaning criteria, scoring rules, and power analysis assumptions are provided in Supplementary material S1–S5. The construct structure, source basis, example items, and scoring approach are summarized in Table 2.

**Table 2:** Scale sources, structure, example items, and scoring.

Variable	Dimensions/items	Main theoretical or scale sources	Example item	Scoring
Teacher caring	Four dimensions, 12 items	Noddings (2012), Teven and McCroskey (1997)	“When I encounter difficulties in learning, teachers give encouragement and support.”	5-point Likert
Academic self-efficacy	Unidimensional, 6 items	Bandura (1977), Zimmerman (2000), Pajares (1996), Chemers et al. (2001)	“Even when the course content is difficult, I am confident that I can gradually master it.”	5-point Likert
Learning engagement	Three dimensions, 9 items	Fredricks et al. (2004), Handelsman et al. (2005), Kahu (2013)	“I actively participate in classroom or course-related learning activities.”	5-point Likert; main analyses used the mean composite score
Perceived course-related learning gains	Unidimensional, 6 items	Pascarella and Terenzini (2005), Kuh (2001)	“Through course learning, I have improved my ability to analyze and solve problems.”	5-point Likert

### 3.3 Control variables

Because students’ perceived course-related learning gains may be influenced by individual background and institutional context, we included gender, year of study, major category, region, and university identifier as control variables in the robustness tests (Carini et al., 2006; Kahu and Nelson, 2018; Schneider and Preckel, 2017). For these variables, the following reference groups were used: female students for gender, first-year students for year of study, humanities and social sciences for major category, central China for region, and U1 for university identifier. The major categories were recoded into humanities and social sciences, science and engineering, business and management, and education and other fields. The baseline model presented the theoretical paths, whereas the robustness model examined whether the core effects remained stable after including these control variables.

### 3.4 Data analysis

The analysis began with data cleaning and sample description, followed by reports of means, standard deviations, skewness, kurtosis, and correlations. To further support the empirical structure of the theory-informed and context-adapted measures, an exploratory factor diagnostic analysis was conducted before interpreting the CFA results. This diagnostic analysis, using the full analytic sample, served as a transparency check rather than a formal scale development procedure. Consequently, it was not used to generate or delete items, or to alter the scoring rules for the main mediation analyses.

Sampling adequacy was evaluated using the Kaiser–Meyer–Olkin (KMO) statistic and Bartlett’s test of sphericity. An oblique rotation method was used due to the theoretical expectation of correlated constructs. Factor retention was determined by parallel analysis, eigenvalues, the scree plot, theoretical interpretability, and the consistency of item loadings with the intended constructs. Reliability was assessed using Cronbach’s alpha, composite reliability (CR), and average variance extracted (AVE). Discriminant validity was also examined using approximate HTMT values derived from item-level correlations among indicators. Subsequently, a four-factor CFA measurement model was estimated using maximum likelihood estimation. Model fit was evaluated using chi-square (χ^2^), degrees of freedom (df), χ^2^/df, CFI, TLI, RMSEA, and SRMR. In the CFA, learning engagement was specified as a single overall latent construct, indicated by all nine behavioral, emotional, and cognitive engagement items. This specification aligned with the composite score mediation strategy and reflected the research focus on the overall engagement mechanism, rather than denying the scale’s theoretically three-dimensional design. Common method bias was assessed using Harman’s single-factor test and a CFA common-method-factor model (Podsakoff et al., 2003). Statistical analyses were performed using SPSS 26.0, Mplus 8.3, and supplementary diagnostic analyses based on the analytic dataset. Specifically, Mplus 8.3 was used for the CFA measurement model and the common-method-factor diagnostic model, while SPSS 26.0 with the PROCESS macro (version 4.0) was used for composite score OLS path analysis and 5,000-resample bootstrap mediation tests. During the hypothesis testing stage, structural paths were estimated as composite score path analyses using standardized composite scores and ordinary least squares (OLS) regression, rather than a full latent variable structural equation model. This strategy was adopted to preserve the theoretically defined scale composites and to ensure that mediation estimates remained directly interpretable after establishing acceptable measurement quality through reliability analysis, the supplementary exploratory diagnostic analysis, and CFA. Therefore, it should not be interpreted or reported as a full latent variable SEM. A 5,000-resample bootstrap procedure was employed to estimate the 95% confidence intervals for specific indirect effects, the sequential indirect effect, and the total indirect effect. Robustness tests further included gender, year of study, major category, region, and university identifier.

## 4 Results

### 4.1 Descriptive statistics and correlations

The means of the four core variables, as reported in Table 3, were all near the midpoint of the scale. Skewness and kurtosis values did not indicate a serious deviation from normality. Correlation analysis revealed significant positive correlations among teacher caring, academic self-efficacy, learning engagement, and perceived course-related learning gains. The correlation between teacher caring and perceived course-related learning gains was r = 0.415. Teacher caring correlated with academic self-efficacy at r = 0.357 and with learning engagement at r = 0.450. The strongest correlation was observed between learning engagement and perceived course-related learning gains (r = 0.529).

**Table 3:** Descriptive statistics and correlation matrix.

Variable	M	SD	Skew	Kurtosis	1	2	3	4
1. Teacher caring	3.127	0.910	−0.134	−0.704	1			
2. Academic self-efficacy	3.061	0.857	−0.091	−0.681	0.357***	1		
3. Learning engagement	3.134	0.946	−0.088	−0.848	0.450***	0.446***	1	
4. Perceived course-related learning gains	3.048	0.897	−0.069	−0.574	0.415***	0.450***	0.529***	1

*N* = 562. ***p < 0.001.

### 4.2 Reliability, convergent validity, and discriminant validity

As shown in Table 4, Cronbach’s alpha values for all core variables exceeded 0.80, CR values exceeded 0.88, and AVE values exceeded 0.50. These results indicate good internal consistency and convergent validity within the current sample. Although the AVE for teacher caring was 0.509, which is close to the conventional 0.50 threshold, it remains within the acceptable range. Combined with its high CR value (0.926) and theoretically consistent standardized factor loadings, this result generally supports the convergent validity of teacher caring. The approximate HTMT results in Table 5 show a maximum value of 0.602, which is below the conservative threshold of 0.85, supporting discriminant validity among the core constructs. The approximate HTMT values were calculated from item-level interconstruct correlations.

**Table 4:** Reliability and convergent validity of the overall constructs.

Variable	Items	Cronbach’s *α*	CR	AVE
Teacher caring	TC_ES1–TC_DG3	0.912	0.926	0.509
Academic self-efficacy	ASE1–ASE6	0.849	0.888	0.570
Learning engagement	LE_BE1–LE_CE3	0.890	0.911	0.533
Perceived course-related learning gains	LG1–LG6	0.867	0.900	0.601

Learning engagement was analyzed as an overall construct in the main model because the hypotheses concerned the overall mediating role of engagement. Although the scale was designed to cover behavioral, emotional, and cognitive dimensions, this table reports the reliability and convergent validity of the overall engagement scale rather than dimension-specific estimates.

**Table 5:** Discriminant validity: approximate HTMT values.

Construct pair	HTMT
Teacher caring/Academic self-efficacy	0.405
Teacher caring/Learning engagement	0.499
Teacher caring/Perceived course-related learning gains	0.467
Academic self-efficacy/Learning engagement	0.513
Academic self-efficacy/Perceived course-related learning gains	0.525
Learning engagement/Perceived course-related learning gains	0.602

### 4.3 Supplementary exploratory factor diagnostic analysis

To further assess the empirical structure of the adapted items, a supplementary exploratory factor analysis was conducted. The data were suitable for factor analysis (KMO = 0.945; Bartlett’s test of sphericity: χ^2^ = 8609.982, df = 528, *p* < 0.001). Parallel analysis supported the retention of four substantive factors: the first four empirical eigenvalues (10.576, 3.352, 2.201, and 1.868) exceeded the corresponding random-data eigenvalues, while the fifth empirical eigenvalue (1.008) did not. The obliquely rotated pattern matrix revealed that items generally loaded on their intended constructs, with no severe cross-loading patterns that would necessitate item deletion or modification of scoring rules. For the nine learning engagement items, a separate one-factor diagnostic analysis showed adequate sampling adequacy (KMO = 0.917; Bartlett’s χ^2^ = 2187.443, df = 36, *p* < 0.001), a dominant first eigenvalue of 4.796 explaining 53.3% of the item variance, and one-factor loadings ranging from 0.628 to 0.756. These results support using an overall learning engagement composite in the mediation model, while still acknowledging the theoretically multidimensional design of engagement. The full exploratory loading matrix is reported in Supplementary Table S6.2.

### 4.4 CFA measurement model

The four-factor CFA measurement model included four latent variables: teacher caring, academic self-efficacy, learning engagement, and perceived course-related learning gains. Learning engagement was specified as a single overall latent factor, indicated by its nine behavioral, emotional, and cognitive engagement items, consistent with the composite score strategy used in the main mediation analysis. The results summarized in Table 6 indicated good model fit: χ^2^ = 875.754, df = 489, χ^2^/df = 1.791, CFI = 0.953, TLI = 0.949, RMSEA = 0.038, and SRMR = 0.034. Standardized factor loadings ranged from 0.630 to 0.757, all within an acceptable range. The standardized CFA measurement path diagram, including standardized factor loadings and standardized error variances, is provided in Supplementary Figure S1.

**Table 6:** CFA measurement model fit indices.

Model	χ^2^	df	χ^2^/df	CFI	TLI	RMSEA	SRMR
Four-factor CFA measurement model	875.754	489	1.791	0.953	0.949	0.038	0.034

This table reports only the CFA measurement model fit indices. The subsequent structural paths were estimated using standardized composite scores and are reported in Tables 8, 9.

Although several standardized factor loadings were slightly below the ideal threshold of 0.70, all loadings exceeded 0.60 and were theoretically consistent with their intended constructs. Additionally, all constructs demonstrated acceptable composite reliability and average variance extracted. Therefore, no item was removed solely based on the 0.70 loading criterion.

Because the hypothesized structural paths were estimated using standardized composite scores rather than a full latent variable structural equation model, no separate global structural model fit indices are reported beyond the CFA measurement model. Structural paths and mediation effects are reported in the subsequent composite score path and bootstrap analyses.

### 4.5 Common method bias

Harman’s single-factor test revealed that the first unrotated factor accounted for 32.05% of the variance, falling below the commonly used 40% criterion. Given Harman’s test is a diagnostic procedure with limited sensitivity, a common-method-factor model using CFA was subsequently estimated. In this diagnostic model, all substantive items were allowed to load onto their theoretical factors and an additional common method factor. The method factor, included solely for diagnostic purposes, aimed to capture the shared variance among the substantive questionnaire items and was compared against the baseline CFA model. As detailed in Table 7, the common-method-factor model exhibited the following fit indices: χ^2^ = 867.793, df = 488, CFI = 0.954, TLI = 0.950, RMSEA = 0.037, and SRMR = 0.035. When compared with the four-factor CFA, the inclusion of the common method factor resulted in only a 0.001 improvement in CFI, which is considerably less than the ΔCFI < 0.01 criterion typically indicating meaningful improvement, and a negligible change in RMSEA. These results indicate that the common-method-factor model did not substantially improve model fit, implying that common method bias is unlikely to substantially compromise the main conclusions. Nevertheless, as all core variables were derived from student self-reports, common method bias cannot be completely ruled out.

**Table 7:** CFA common-method-factor test.

Model	χ^2^	df	CFI	TLI	RMSEA	SRMR	Comparison with CFA
Four-factor CFA	875.754	489	0.953	0.949	0.038	0.034	Baseline model
Common-method-factor model	867.793	488	0.954	0.950	0.037	0.035	ΔCFI = 0.001

### 4.6 Path analysis

The baseline composite score path analysis was performed using standardized composite scores and OLS regression, with standardized regression path coefficients presented in Table 8. The results indicated a positive and statistically significant total association between teacher caring and perceived course-related learning gains (*β* = 0.415, R^2^ = 0.173, *p* < 0.001), thus supporting H1. Teacher caring was positively associated with academic self-efficacy (*β* = 0.357, *p* < 0.001). In the regression model for learning engagement, both teacher caring and academic self-efficacy demonstrated positive unique associations with learning engagement, with *β* = 0.333 and *β* = 0.327, respectively (*p* < 0.001). When teacher caring, academic self-efficacy, and learning engagement were simultaneously entered into the regression model for perceived course-related learning gains, all three variables exhibited positive unique associations with perceived gains: teacher caring (*β* = 0.177), academic self-efficacy (*β* = 0.233), and learning engagement (*β* = 0.346). This model accounted for 36.1% of the variance in perceived course-related learning gains.

**Table 8:** Standardized regression path analysis.

Regression model/dependent variable (R^2^)	Independent variable/path	Standardized β	*p*
Total-effect model: perceived course-related learning gains (R^2^ = 0.173)	Teacher caring → perceived course-related learning gains	0.415	< 0.001
M1: Academic self-efficacy (R^2^ = 0.127)	Teacher caring → Academic self-efficacy	0.357	< 0.001
M2: Learning engagement (R^2^ = 0.296)	Teacher caring → Learning engagement	0.333	< 0.001
M2: Learning engagement (R^2^ = 0.296)	Academic self-efficacy → Learning engagement	0.327	< 0.001
M3: Perceived course-related learning gains (R^2^ = 0.361)	Teacher caring → perceived course-related learning gains (controlling for mediators)	0.177	< 0.001
M3: Perceived course-related learning gains (R^2^ = 0.361)	Academic self-efficacy → perceived course-related learning gains	0.233	< 0.001
M3: Perceived course-related learning gains (R^2^ = 0.361)	Learning engagement → perceived course-related learning gains	0.346	< 0.001

R^2^ is shown in the model/dependent-variable column and refers to the explained variance of the corresponding dependent variable in each regression model.

### 4.7 Mediation effects

**Figure 2:**
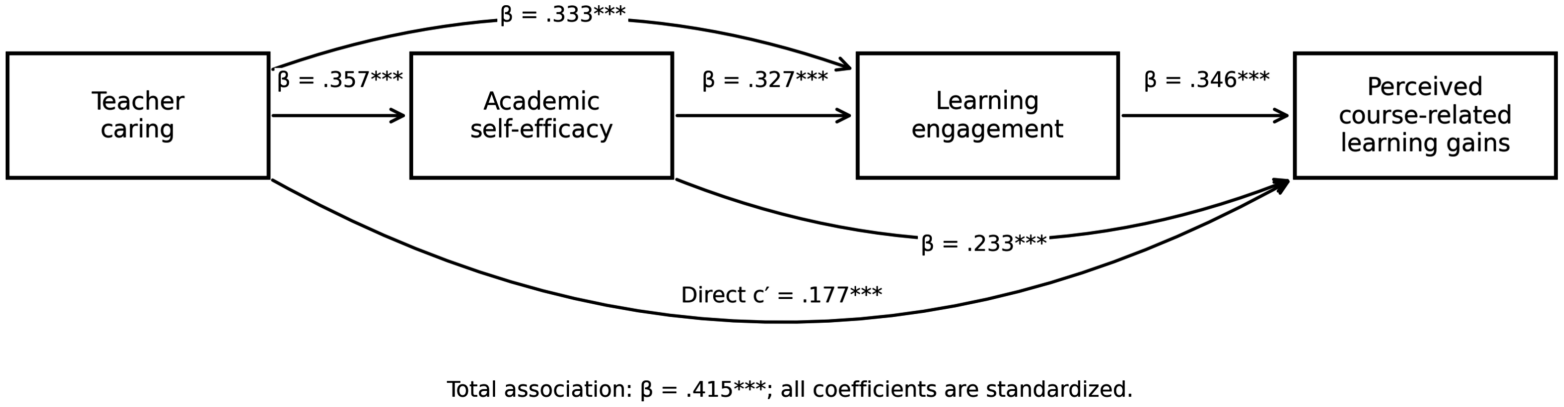
Final standardized composite score path model. Coefficients are standardized OLS path coefficients from the baseline composite score model. ***p < 0.001. The direct path is reported after academic self-efficacy and learning engagement are included in the model; the total association between teacher caring and perceived course-related learning gains was β = 0.415, *p* < 0.001.

The bootstrap results presented in Table 9 indicate that the indirect association from teacher caring to perceived course-related learning gains through academic self-efficacy was 0.083 (95% CI [0.053, 0.117]). The indirect association through learning engagement was 0.116 (95% CI [0.081, 0.154]). The sequential indirect association from teacher caring to perceived course-related learning gains, mediated first by academic self-efficacy and then by learning engagement, was 0.040 (95% CI [0.026, 0.057]). Since none of the confidence intervals included zero, hypotheses H2, H3, and H4 are supported. The direct path remained significant, suggesting that academic self-efficacy and learning engagement act as partial mediators in the composite score mediation model (Figure 2).

**Table 9:** Bootstrap mediation effects.

Effect path	Standardized effect	95% CI
Teacher caring → Academic self-efficacy → perceived course-related learning gains	0.083	[0.053, 0.117]
Teacher caring → Learning engagement → perceived course-related learning gains	0.116	[0.081, 0.154]
Teacher caring → Academic self-efficacy → Learning engagement → perceived course-related learning gains	0.040	[0.026, 0.057]
Total indirect effect	0.239	[0.188, 0.293]
Direct effect c′	0.177	[0.101, 0.254]

### 4.8 Robustness tests: model with control variables

To further examine the robustness of the findings, gender, year of study, major category, region, and university identifier were included as control variables. As summarized in Tables 10, 11, the key associations linking teacher caring with academic self-efficacy, learning engagement, and perceived course-related learning gains remained statistically significant. The associations of academic self-efficacy and learning engagement with perceived course-related learning gains also remained stable. Compared with the baseline model, the core coefficients changed only slightly, suggesting that differences in sample background did not alter the main conclusions. Because the control variables were included as robustness adjustments rather than substantive predictors, their individual coefficients are not interpreted in detail; importantly, the inclusion of major category and university identifier did not change the interpretation of the focal paths.

**Table 10:** Key path results after including control variables.

Model	Independent variable/path	Standardized β	R^2^	*p*
M1	Teacher caring → Academic self-efficacy	0.369	0.143	< 0.001
M2	Teacher caring → Learning engagement	0.342	0.306	< 0.001
M2	Academic self-efficacy → Learning engagement	0.326	0.306	< 0.001
M3	Teacher caring → perceived course-related learning gains	0.177	0.373	< 0.001
M3	Academic self-efficacy → perceived course-related learning gains	0.229	0.373	< 0.001
M3	Learning engagement → perceived course-related learning gains	0.349	0.373	< 0.001

Control variables include gender, year of study, major category, region, and university identifier. All coefficients are standardized. R^2^ refers to the explained variance of the corresponding dependent variable in each regression model.

**Table 11:** Bootstrap mediation effects after including control variables.

Effect path	Standardized effect	95% CI
Teacher caring → Academic self-efficacy → perceived course-related learning gains	0.084	[0.052, 0.120]
Teacher caring → Learning engagement → perceived course-related learning gains	0.119	[0.085, 0.159]
Teacher caring → Academic self-efficacy → Learning engagement → perceived course-related learning gains	0.042	[0.027, 0.059]
Total indirect effect	0.246	[0.194, 0.300]
Direct effect c′	0.177	[0.099, 0.255]

## 5 Discussion

This study examined how teacher caring is associated with Chinese undergraduates’ perceived course-related learning gains through academic self-efficacy and learning engagement. Overall, the findings support the proposed sequential mediation model: students who perceived stronger teacher caring tended to report stronger academic self-efficacy, higher learning engagement, and greater course-related learning gains. Because the study used cross-sectional self-report data, these findings should be understood as statistical associations consistent with a relational–motivational pathway rather than as evidence of causal ordering.

The findings help clarify the psychological process through which external interpersonal care may be internalized into learning beliefs and then expressed as sustained learning participation. From the perspective of caring theory, teacher caring communicates that students’ difficulties are noticed, their efforts are valued, and their development is taken seriously. Such relational cues may reduce students’ perceived threat in learning situations and make feedback easier to interpret as constructive support rather than as judgment. In this sense, caring does not operate only as emotional warmth; it also functions as a learning-relevant relational condition that can support students’ confidence in handling course tasks.

Social cognitive theory provides a further explanation for the first part of the sequence. Teacher encouragement, specific feedback, respect, and developmental guidance may be associated with stronger academic self-efficacy because they provide students with information about how to improve, reinforce the belief that learning difficulties are manageable, and make academic progress appear attainable. This is particularly relevant in Chinese higher education, where students often face strong achievement expectations, relatively teacher-centered classroom traditions, and high sensitivity to evaluative feedback. In such a context, caring teacher behaviors may help students reinterpret teacher authority as accessible support, thereby strengthening confidence in course learning.

Learning engagement explains the next part of the process. Students with stronger academic self-efficacy are more likely to invest effort, maintain positive emotions, and use cognitive strategies when facing course tasks. Engagement then becomes the process through which capability beliefs are translated into perceived gains in knowledge, problem-solving, autonomous learning, and developmental clarity. The slightly larger mediating effect of learning engagement indicates that learning gains are closely tied to students’ active participation in coursework rather than to relational support or confidence alone. The significant sequential indirect association, therefore, suggests a conversion process from supportive teacher–student relationships, to psychological resources, to learner role investment, and finally to perceived course-related development. The remaining direct association between teacher caring and perceived learning gains also suggests that other mechanisms, such as belonging, learning meaning, classroom safety, feedback quality, and help-seeking accessibility, may complement the two mediators examined here.

### 5.1 Theoretical implications

The findings offer several theoretical implications for understanding teacher caring in higher education.

First, this study extends teacher caring research by shifting the focus from whether teacher caring is associated with learning outcomes to how such caring is linked to students’ learning beliefs, engagement, much like perceived developmental gains. Prior research has often treated supportive teacher–student relationships as a broad contextual resource. The present model specifies a more fine-grained process: caring is linked to perceived gains partly because it is associated with students’ confidence in learning and with their behavioral, emotional, and cognitive investment in coursework. This perspective suggests that the educational value of teacher caring lies not only in emotional warmth but also in its connection with motivational beliefs and learning behaviors.

Second, the study connects social cognitive theory with learning engagement theory. Academic self-efficacy reflects students’ beliefs about whether they can organize and execute learning actions, whereas learning engagement reflects the extent to which they actually invest effort, emotion, and cognitive resources in learning tasks. The significant sequential path indicates that capability beliefs and engagement should not be treated as isolated mechanisms. Instead, confidence appears to be most relevant to learning gains when it is accompanied by active engagement in coursework.

Third, the study uses a supportive relationship perspective as a supplementary bridge between educational psychology and organizational psychology. In this framework, teacher caring can be viewed as a proximal support resource, academic self-efficacy as a learning-related psychological resource, and learning engagement as students’ investment in the learner role. This cross-disciplinary lens helps connect teacher–student relationship research with broader discussions of supportive learning environments, role engagement, and developmental outcomes. It also suggests that higher education teaching quality should be understood as a relational and motivational process, not only as content delivery or assessment design.

### 5.2 Practical implications

The results also have practical implications for teaching improvement, student support, and quality evaluation in universities.

First, faculty development should translate teacher caring into observable and trainable teaching behaviors. Teacher caring should not be limited to a friendly attitude; it can be expressed through timely feedback, specific guidance, respect for student expression, encouragement to try, and developmental advice. Through classroom interaction, assignment feedback, consultation, and learning guidance, teachers can make support more visible and useful to students.

Second, student support systems should pay closer attention to academic self-efficacy. For students who experience learning difficulties, simply providing more resources or stricter requirements may not be enough. Universities can help students build confidence through staged achievement feedback, peer support, academic tutoring, growth-oriented evaluation, and opportunities to experience gradual progress.

Third, efforts to improve learning engagement should address behavioral, emotional, and cognitive dimensions together. Task completion and attendance are important, but they do not fully represent deep engagement. Teachers can use problem-driven learning, authentic tasks, collaborative inquiry, reflective assessment, and meaningful feedback to guide students from completing requirements toward active understanding, strategy use, and knowledge transfer.

Finally, university-level quality evaluation may benefit from incorporating process-oriented indicators such as perceived teacher caring, academic self-efficacy, and learning engagement. Compared with relying only on final grades or satisfaction ratings, these indicators can help identify weak points in the learning support chain and provide evidence for course improvement, faculty training, and student development services.

### 5.3 Limitations and future directions

Several limitations should be considered when interpreting the findings; these also point to directions for future research.

First, the study used a cross-sectional design. Although the proposed mediation model followed a theoretically ordered sequence, the data cannot establish temporal order or causal mechanisms. Future studies could use longitudinal tracking, cross-lagged models, intervention studies, or experimental designs to examine how teacher caring, academic self-efficacy, learning engagement, and learning gains unfold over time. Second, all core variables were measured through student self-report questionnaires, which may involve common method bias and subjective evaluation bias. Although diagnostic tests did not indicate a serious common method problem, future research should incorporate multi-source data, such as classroom observations, learning platform records, teacher evaluations, course grades, or portfolio assessments.

Third, the structural paths and mediation effects were estimated with standardized composite scores using OLS regression rather than a full latent variable structural equation model. This approach makes the results easy to interpret, but it does not explicitly account for measurement error in the structural paths. Future studies could replicate the model using latent variable SEM and further test whether learning engagement functions as a second-order construct composed of behavioral, emotional, and cognitive engagement. Relatedly, this study analyzed learning engagement as an overall construct and did not compare the specific roles of its three dimensions. Future research could examine whether behavioral, emotional, and cognitive engagement play distinct mediating roles. Because the supplementary exploratory analysis was conducted with the same analytic sample rather than an independent scale development sample, the measurement evidence should be interpreted as study-specific validation evidence rather than as full validation of a standalone psychometric scale.

Fourth, the sample consisted of Chinese undergraduate students, and participating universities were represented only by anonymized identifiers. The generalizability of the findings, therefore, requires further testing across regions, institution types, disciplines, and educational systems. Finally, perceived course-related learning gains capture students’ subjective sense of development in knowledge, competence, learning strategies, and developmental clarity. While meaningful for understanding the learning experience, this differs from objective indicators of achievement. Future research could combine perceived learning gains with objective or performance-based measures to provide a more comprehensive evaluation of how teacher caring is linked to learning quality.

## 6 Conclusion

This study aimed to clarify not only whether teacher caring is associated with undergraduate students’ perceived course-related learning gains, but also how this association may operate. Based on data from 562 Chinese undergraduates, the results showed that students who perceived higher levels of teacher caring also reported stronger academic self-efficacy, greater learning engagement, and higher perceived course-related learning gains. Academic self-efficacy and learning engagement functioned as both separate and sequential mediators, suggesting that teacher caring is linked to learning gains through a process in which relational support is associated with students’ confidence in learning and their active investment in coursework. Although the cross-sectional and self-report design does not allow for causal conclusions, the findings provide evidence consistent with a relational–motivational pathway from teacher caring to students’ perceived learning development. The study, therefore, extends teacher caring research by shifting the focus from whether teacher caring is associated with learning outcomes to how such caring is linked to students’ learning beliefs, engagement, and perceived developmental gains. For practice, the findings suggest that efforts to improve teaching quality should not be limited to monitoring final outcomes. Universities and teachers should also cultivate caring instructional relationships that help students build confidence, stay engaged, and experience meaningful growth through coursework.

## Data Availability

The original contributions presented in the study are included in the article/Supplementary material, further inquiries can be directed to the corresponding author.
