# Supplementary material for: Linking teacher caring to perceived course-related learning gains: the sequential mediating roles of academic self-efficacy and learning engagement
Source: Front Psychol. 2026 Jul 16;17:1901610. doi: 10.3389/fpsyg.2026.1901610 (PMC13422389; doi:10.3389/fpsyg.2026.1901610)
Supplement: Supplementary file 1 [file Supplementary_file_1.DOCX]

**Supplementary Material**

**Survey Instrument, Scoring Rules, and Data-Cleaning Criteria**

The survey was administered in Chinese. The English wording below is a reporting translation of the Chinese item text used in the analysis codebook. All substantive items were rated on a five-point Likert scale, where 1 = strongly disagree and 5 = strongly agree. Perceived course-related learning gains were measured as students' perceived gains during course learning rather than as objective grades.

**S1. Demographic and Control Variables**

| **Variable** | **Categories / Coding** | **Use in main analysis** |
| --- | --- | --- |
| Gender | Female; Male | Control variable; female served as the reference group. |
| Year of study | Year 1; Year 2; Year 3; Year 4 | Control variable; Year 1 served as the reference group. |
| Major category | Humanities and social sciences; Science and engineering; Business and management; Education and other fields | Control variable; humanities and social sciences served as the reference group. |
| Region | Central China; Eastern China | Control variable; Central China served as the reference group. |
| University identifier | U1-U6 | Control variable; U1 served as the reference group. Identifiers were anonymized. |

**S2. Measurement Items**

| **Variable** | **Construct / Dimension** | **English reporting translation** |
| --- | --- | --- |
| TC_ES1 | Teacher caring - emotional support | Teachers are able to notice my emotions and state during learning. |
| TC_ES2 | Teacher caring - emotional support | When I encounter difficulties in learning, teachers give encouragement and support. |
| TC_ES3 | Teacher caring - emotional support | Teachers make me feel cared for in classroom learning. |
| TC_AS1 | Teacher caring - academic support | Teachers provide specific guidance for my learning problems. |
| TC_AS2 | Teacher caring - academic support | The feedback given by teachers helps me improve my learning methods. |
| TC_AS3 | Teacher caring - academic support | Teachers help me clarify the key points and directions for improvement in learning tasks. |
| TC_RI1 | Teacher caring - respect and inclusion | Teachers respect my views and expressions in classroom interactions. |
| TC_RI2 | Teacher caring - respect and inclusion | Teachers treat students with different academic foundations and backgrounds fairly. |
| TC_RI3 | Teacher caring - respect and inclusion | Teachers accept students’ different ways of understanding problems. |
| TC_DG1 | Teacher caring - developmental guidance | Teachers encourage me to connect current learning with future development. |
| TC_DG2 | Teacher caring - developmental guidance | Teachers guide me to think about my learning goals and growth direction. |
| TC_DG3 | Teacher caring - developmental guidance | Teachers make me believe that sustained effort can bring long-term progress. |
| ASE1 | Academic self-efficacy | I believe I can complete the main learning tasks in the course. |
| ASE2 | Academic self-efficacy | Even when the course content is difficult, I am confident that I can gradually master it. |
| ASE3 | Academic self-efficacy | I can find ways to solve learning problems through effort. |
| ASE4 | Academic self-efficacy | When facing examinations or course assessments, I believe I can prepare well. |
| ASE5 | Academic self-efficacy | When learning does not progress smoothly, I can still maintain confidence in completing the task. |
| ASE6 | Academic self-efficacy | I believe I have the ability to achieve the expected learning goals. |
| LE_BE1 | Learning engagement - behavioral engagement | I complete course learning tasks on time. |
| LE_BE2 | Learning engagement - behavioral engagement | I actively participate in classroom or course-related learning activities. |
| LE_BE3 | Learning engagement - behavioral engagement | Even without external requirements, I invest time in learning. |
| LE_EE1 | Learning engagement - emotional engagement | I maintain a high level of interest in course learning. |
| LE_EE2 | Learning engagement - emotional engagement | I experience positive emotions during the learning process. |
| LE_EE3 | Learning engagement - emotional engagement | I think course learning is meaningful to me. |
| LE_CE1 | Learning engagement - cognitive engagement | I actively think about the connections among knowledge points. |
| LE_CE2 | Learning engagement - cognitive engagement | I try to use different methods to understand complex content. |
| LE_CE3 | Learning engagement - cognitive engagement | I reflect on my learning strategies and make adjustments. |
| LG1 | Perceived course-related learning gains | Through course learning, I have mastered more professional knowledge. |
| LG2 | Perceived course-related learning gains | Through course learning, I have improved my ability to analyze and solve problems. |
| LG3 | Perceived course-related learning gains | Through course learning, I have improved my learning methods. |
| LG4 | Perceived course-related learning gains | Through course learning, I have become clearer about my future learning direction. |
| LG5 | Perceived course-related learning gains | Through course learning, my autonomous learning ability has improved. |
| LG6 | Perceived course-related learning gains | Overall, I think I have achieved clear learning growth. |

**S3. Attention Checks and Data-Cleaning Criteria**

For reporting purposes, exclusion categories were coded hierarchically and reported as mutually exclusive categories. If a response met multiple exclusion criteria, it was counted only once according to the first applicable criterion in the cleaning sequence.

| **Rule** | **Criterion** | **Action** | **Rationale** |
| --- | --- | --- | --- |
| Failed both attention checks | ATT1 != 4 AND ATT2 != 2 | Exclude | Direct exclusion required failure on both attention-check items. |
| Failed one attention-check item | ATT1 != 4 XOR ATT2 != 2 | Review with other quality indicators | A single failed attention-check item was not used as a sole exclusion criterion. |
| Overly fast completion | Duration_sec < 120 | Exclude | Insufficient time to read all questionnaire items. |
| Straight-lining | Same option across nearly all core items | Exclude | Low-effort response pattern. |
| Random response pattern | Very inconsistent pattern with failed quality checks | Exclude | Likely careless response. |
| Near-duplicate responses | Highly similar response pattern to another case plus metadata warning | Review / Exclude | Potential duplicate submission. |
| Extreme but consistent responses | Very high or very low scores but passed quality checks | Keep / Review | Extreme attitudes may be valid and were not removed automatically. |
| Exclusion-category reporting | Hierarchical, mutually exclusive coding of exclusion reasons | Report once | Prevents double-counting when one response meets more than one exclusion criterion. |

**S4. Scoring Rules**

| **Score** | **Items** | **Interpretation** |
| --- | --- | --- |
| Teacher caring total score | Mean of all 12 teacher-caring items: TC_ES1-TC_ES3, TC_AS1-TC_AS3, TC_RI1-TC_RI3, and TC_DG1-TC_DG3. | Higher scores indicate stronger perceived teacher caring. |
| Teacher caring dimensions | Emotional support = mean of TC_ES1-TC_ES3; academic support = mean of TC_AS1-TC_AS3; respect and inclusion = mean of TC_RI1-TC_RI3; developmental guidance = mean of TC_DG1-TC_DG3. | Dimension scores can be used for descriptive or profile-based analyses. |
| Academic self-efficacy | Mean of ASE1-ASE6. | Higher scores indicate stronger confidence in completing learning tasks. |
| Learning engagement | Mean of all nine learning-engagement items: LE_BE1-LE_BE3, LE_EE1-LE_EE3, and LE_CE1-LE_CE3. | Higher scores indicate stronger behavioral, emotional, and cognitive engagement. |
| Learning engagement dimensions | Behavioral engagement = mean of LE_BE1-LE_BE3; emotional engagement = mean of LE_EE1-LE_EE3; cognitive engagement = mean of LE_CE1-LE_CE3. | Dimension scores can be reported in supplementary analyses. |
| Perceived course-related learning gains | Mean of LG1-LG6. | Higher scores indicate stronger perceived course-related learning gains. |

**S5. Power Analysis Assumptions**

A sample-size justification for the sequential mediation model was conducted using an a priori power-analysis framework for mediation models. The calculation did not use the sample-specific path coefficients from the final dataset. Instead, conservative small standardized component paths were assumed for the focal sequential indirect association. The target was 80% power at α = .05 for detecting the sequential indirect association using a two-sided 95% confidence interval.

| **Parameter** | **Assumed value** | **Rationale / interpretation** |
| --- | --- | --- |
| Teacher caring → Academic self-efficacy (a1) | .15 | Conservative small standardized component path |
| Academic self-efficacy → Learning engagement (d21) | .15 | Conservative small standardized component path |
| Learning engagement → Perceived course-related learning gains (b2) | .15 | Conservative small standardized component path |
| Sequential indirect association (a1 × d21 × b2) | .0034 | Focal effect for the sequential mediation path |
| Other direct paths | .10–.15 | Small values specified for remaining model paths |
| Target power | .80 | Conventional minimum power criterion |
| Alpha level | .05 | Two-sided 95% confidence interval criterion |
| Minimum required sample size | N ≈ 540 | Monte Carlo simulation estimate |
| Final analytic sample | N = 562 | Exceeds the estimated minimum requirement |

Under these conservative assumptions, the final analytic sample of N = 562 exceeded the estimated minimum requirement of approximately N = 540, thereby supporting the adequacy of the sample size for detecting the focal sequential indirect association.

**S6. Supplementary Exploratory Factor Diagnostic Analysis**

A supplementary exploratory factor diagnostic analysis was conducted to examine whether the theory-informed and context-adapted items showed an empirical structure broadly consistent with the theoretically specified constructs. This analysis was not used to develop a new scale, delete items, or change the scoring rules; instead, it served as an additional transparency check before interpreting the CFA measurement model and the composite-score mediation analyses.

**Supplementary Table S6.1. Summary of Exploratory Factor Diagnostic Results**

| **Index** | **Result** | **Interpretation** |
| --- | --- | --- |
| Analytic sample | N = 562 | Valid responses used in the main analysis |
| Number of substantive items | 33 | Teacher caring = 12; academic self-efficacy = 6; learning engagement = 9; perceived learning gains = 6 |
| KMO | 0.945 | Excellent sampling adequacy |
| Bartlett’s test of sphericity | χ² = 8609.982, df = 528, p < .001 | The item correlation matrix was factorable |
| Factor retention | Four substantive factors | Parallel analysis, eigenvalues, scree-plot inspection, and theoretical interpretability supported the four overall constructs used in the main model |
| Learning engagement diagnostic | KMO = 0.917; Bartlett’s χ² = 2187.443, df = 36, p < .001; first eigenvalue = 4.796; one-factor loading range = 0.628-0.756 | Supported the empirical plausibility of using an overall learning-engagement composite while retaining the theoretical three-component design |

Note. The exploratory factor analysis was conducted as a diagnostic transparency check rather than as a formal scale-development procedure. The four-factor exploratory solution was rotated using an oblique rotation because the constructs were theoretically expected to be correlated.

**Supplementary Table S6.2. Obliquely Rotated Exploratory Pattern Matrix**

| **Item** | **Intended construct** | **F1 Teacher caring** | **F2 Academic self-efficacy** | **F3 Learning engagement** | **F4 Learning gains** | **Primary factor** | **Primary loading** | **Highest cross-loading** |
| --- | --- | --- | --- | --- | --- | --- | --- | --- |
| TC_ES1 | Teacher caring | 0.695 | 0.035 | -0.034 | -0.05 | TC | 0.695 | 0.05 |
| TC_ES2 | Teacher caring | 0.7 | 0.029 | -0.027 | -0.013 | TC | 0.7 | 0.029 |
| TC_ES3 | Teacher caring | 0.734 | -0.002 | -0.044 | -0.03 | TC | 0.734 | 0.044 |
| TC_AS1 | Teacher caring | 0.612 | -0.03 | 0.064 | 0.026 | TC | 0.612 | 0.064 |
| TC_AS2 | Teacher caring | 0.692 | -0.033 | 0.029 | 0.018 | TC | 0.692 | 0.033 |
| TC_AS3 | Teacher caring | 0.679 | 0.008 | 0.022 | -0.035 | TC | 0.679 | 0.035 |
| TC_RI1 | Teacher caring | 0.652 | 0.014 | 0.026 | 0.03 | TC | 0.652 | 0.03 |
| TC_RI2 | Teacher caring | 0.675 | -0.021 | 0.009 | 0.015 | TC | 0.675 | 0.021 |
| TC_RI3 | Teacher caring | 0.703 | -0.021 | -0.071 | 0.071 | TC | 0.703 | 0.071 |
| TC_DG1 | Teacher caring | 0.672 | -0.003 | 0.078 | -0.051 | TC | 0.672 | 0.078 |
| TC_DG2 | Teacher caring | 0.657 | 0.014 | 0.015 | 0.034 | TC | 0.657 | 0.034 |
| TC_DG3 | Teacher caring | 0.667 | 0.025 | -0.01 | 0.001 | TC | 0.667 | 0.025 |
| ASE1 | Academic self-efficacy | -0.027 | 0.618 | 0.035 | 0.042 | ASE | 0.618 | 0.042 |
| ASE2 | Academic self-efficacy | -0.003 | 0.638 | 0.009 | 0.049 | ASE | 0.638 | 0.049 |
| ASE3 | Academic self-efficacy | -0.053 | 0.712 | 0.029 | 0.018 | ASE | 0.712 | 0.053 |
| ASE4 | Academic self-efficacy | 0.036 | 0.72 | 0.023 | -0.031 | ASE | 0.72 | 0.036 |
| ASE5 | Academic self-efficacy | 0.031 | 0.743 | -0.056 | -0.001 | ASE | 0.743 | 0.056 |
| ASE6 | Academic self-efficacy | 0.019 | 0.711 | -0.032 | -0.031 | ASE | 0.711 | 0.032 |
| LE_BE1 | Learning engagement | -0.029 | 0.032 | 0.67 | -0.018 | LE | 0.67 | 0.032 |
| LE_BE2 | Learning engagement | 0.003 | 0.055 | 0.66 | -0.019 | LE | 0.66 | 0.055 |
| LE_BE3 | Learning engagement | 0.027 | 0.017 | 0.591 | 0.027 | LE | 0.591 | 0.027 |
| LE_EE1 | Learning engagement | 0.012 | 0.004 | 0.683 | 0.014 | LE | 0.683 | 0.014 |
| LE_EE2 | Learning engagement | 0.023 | 0.025 | 0.697 | -0.038 | LE | 0.697 | 0.038 |
| LE_EE3 | Learning engagement | -0.013 | 0.037 | 0.672 | -0.004 | LE | 0.672 | 0.037 |
| LE_CE1 | Learning engagement | -0.015 | -0.084 | 0.728 | 0.034 | LE | 0.728 | 0.084 |
| LE_CE2 | Learning engagement | -0.019 | -0.031 | 0.664 | 0.092 | LE | 0.664 | 0.092 |
| LE_CE3 | Learning engagement | 0.02 | -0.023 | 0.784 | -0.043 | LE | 0.784 | 0.043 |
| LG1 | Perceived course-related learning gains | 0.01 | 0.002 | 0.046 | 0.648 | LG | 0.648 | 0.046 |
| LG2 | Perceived course-related learning gains | 0.03 | 0.023 | -0.057 | 0.675 | LG | 0.675 | 0.057 |
| LG3 | Perceived course-related learning gains | -0.025 | 0.005 | 0.003 | 0.753 | LG | 0.753 | 0.025 |
| LG4 | Perceived course-related learning gains | -0.017 | -0.008 | 0.081 | 0.699 | LG | 0.699 | 0.081 |
| LG5 | Perceived course-related learning gains | -0.016 | 0.025 | -0.056 | 0.792 | LG | 0.792 | 0.056 |
| LG6 | Perceived course-related learning gains | 0.043 | -0.037 | 0.027 | 0.723 | LG | 0.723 | 0.043 |

Note. Primary loadings are shown in the column corresponding to each item’s intended construct. The largest cross-loading for each item was below .10 in this diagnostic solution, indicating no severe cross-loading pattern requiring item deletion or rescoring.

**Supplementary Table S6.3. One-Factor Diagnostic Results for Learning Engagement Items**

| **Item** | **Construct** | **One-factor PAF loading** | **Communality** |
| --- | --- | --- | --- |
| LE_BE1 | Learning engagement | 0.665 | 0.442 |
| LE_BE2 | Learning engagement | 0.681 | 0.464 |
| LE_BE3 | Learning engagement | 0.628 | 0.394 |
| LE_EE1 | Learning engagement | 0.698 | 0.487 |
| LE_EE2 | Learning engagement | 0.701 | 0.491 |
| LE_EE3 | Learning engagement | 0.683 | 0.467 |
| LE_CE1 | Learning engagement | 0.697 | 0.485 |
| LE_CE2 | Learning engagement | 0.688 | 0.474 |
| LE_CE3 | Learning engagement | 0.756 | 0.571 |

Note. This learning-engagement diagnostic analysis supports use of an overall engagement composite in the main mediation model. It does not imply that behavioral, emotional, and cognitive engagement are theoretically identical; rather, it indicates that the nine items also share a sufficient common engagement component for the present study’s global mediation analysis.

**S7. CFA Measurement Model Path Diagram**

The four-factor CFA measurement model showed good fit: χ² = 875.754, df = 489, χ²/df = 1.791, CFI = 0.953, TLI = 0.949, RMSEA = 0.038, and SRMR = 0.034. Standardized factor loadings ranged from 0.630 to 0.757, and standardized error variances ranged from 0.427 to 0.603.


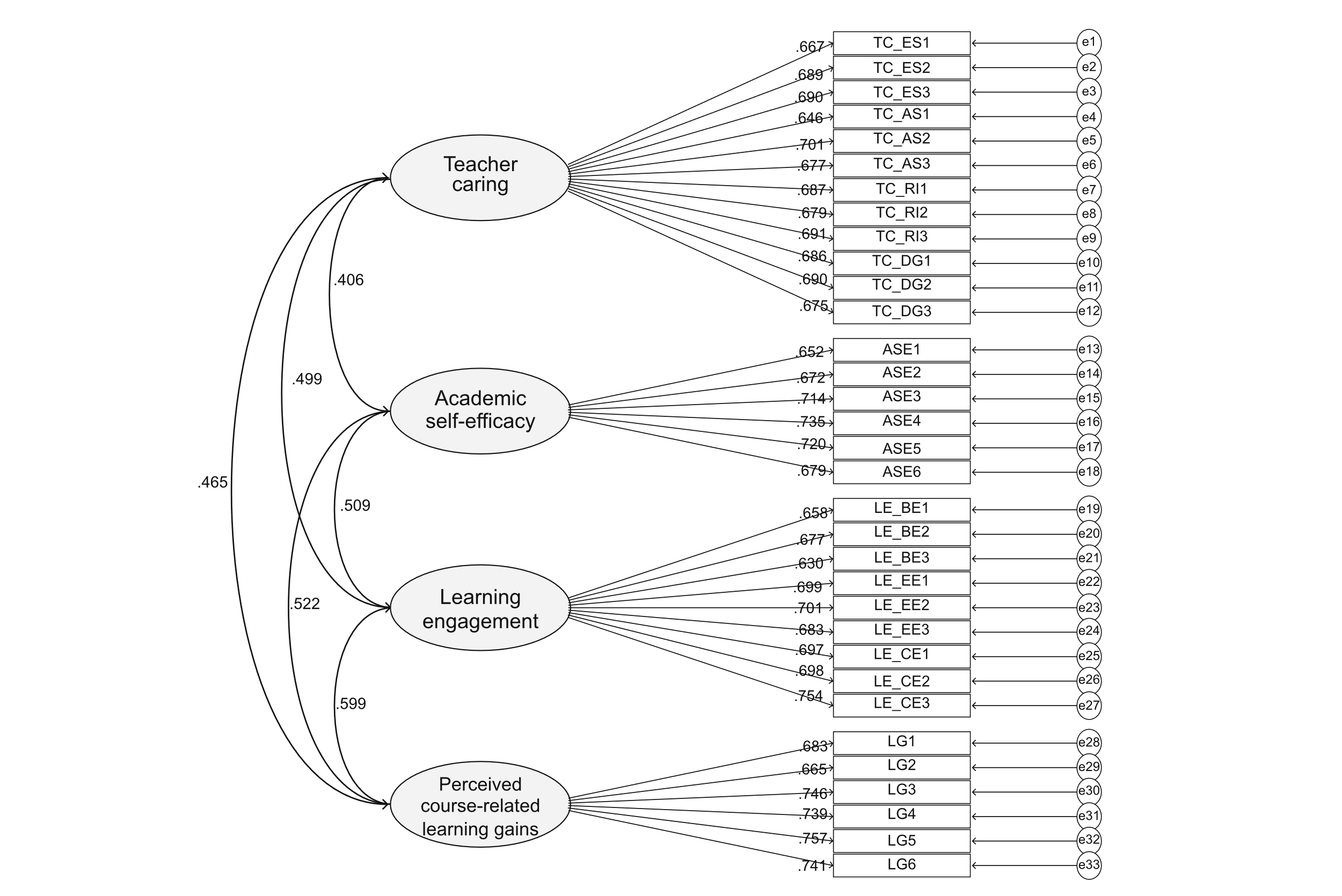


**Supplementary Figure S1. CFA Measurement Model With Standardized Factor Loadings and Error Terms**

Note. Standardized factor loadings are displayed on arrows from latent variables to observed indicators. Standardized error variances are displayed next to each observed indicator. Dashed lines represent latent-factor correlations.

**Supplementary Table S7.1. CFA Standardized Factor Loadings and Error Terms**

| **Item** | **Latent factor** | **Standardized loading** | **Standardized error variance** |
| --- | --- | --- | --- |
| TC_ES1 | Teacher caring | 0.667 | 0.556 |
| TC_ES2 | Teacher caring | 0.689 | 0.525 |
| TC_ES3 | Teacher caring | 0.69 | 0.523 |
| TC_AS1 | Teacher caring | 0.646 | 0.582 |
| TC_AS2 | Teacher caring | 0.701 | 0.509 |
| TC_AS3 | Teacher caring | 0.677 | 0.541 |
| TC_RI1 | Teacher caring | 0.687 | 0.527 |
| TC_RI2 | Teacher caring | 0.679 | 0.539 |
| TC_RI3 | Teacher caring | 0.691 | 0.522 |
| TC_DG1 | Teacher caring | 0.686 | 0.53 |
| TC_DG2 | Teacher caring | 0.69 | 0.525 |
| TC_DG3 | Teacher caring | 0.675 | 0.544 |
| ASE1 | Academic self-efficacy | 0.652 | 0.574 |
| ASE2 | Academic self-efficacy | 0.672 | 0.549 |
| ASE3 | Academic self-efficacy | 0.714 | 0.491 |
| ASE4 | Academic self-efficacy | 0.735 | 0.46 |
| ASE5 | Academic self-efficacy | 0.72 | 0.482 |
| ASE6 | Academic self-efficacy | 0.679 | 0.539 |
| LE_BE1 | Learning engagement | 0.658 | 0.567 |
| LE_BE2 | Learning engagement | 0.677 | 0.542 |
| LE_BE3 | Learning engagement | 0.63 | 0.603 |
| LE_EE1 | Learning engagement | 0.699 | 0.512 |
| LE_EE2 | Learning engagement | 0.701 | 0.509 |
| LE_EE3 | Learning engagement | 0.683 | 0.533 |
| LE_CE1 | Learning engagement | 0.697 | 0.515 |
| LE_CE2 | Learning engagement | 0.698 | 0.512 |
| LE_CE3 | Learning engagement | 0.754 | 0.432 |
| LG1 | Perceived course-related learning gains | 0.683 | 0.534 |
| LG2 | Perceived course-related learning gains | 0.665 | 0.558 |
| LG3 | Perceived course-related learning gains | 0.746 | 0.443 |
| LG4 | Perceived course-related learning gains | 0.739 | 0.453 |
| LG5 | Perceived course-related learning gains | 0.757 | 0.427 |
| LG6 | Perceived course-related learning gains | 0.741 | 0.45 |
